# FKBP51 decreases cell proliferation and increases progestin sensitivity of human endometrial adenocarcinomas by inhibiting Akt

**DOI:** 10.18632/oncotarget.18903

**Published:** 2017-06-30

**Authors:** Jing Dong, Yulian Jiao, Wenli Mu, Bingru Lu, Muyun Wei, Linying Sun, Shengnan Hu, Bin Cui, Xiaowen Liu, Zijiang Chen, Yueran Zhao

**Affiliations:** ^1^ Department of Central Lab, Shandong Provincial Hospital Affiliated to Shandong University, Jinan, Shandong, China; ^2^ Reproductive Hospital affiliated to Shandong University, Jinan, Shandong, China

**Keywords:** Akt, endometrial adenocarcinoma, FKBP51, progestin sensitivity, proliferation

## Abstract

In this study, we investigated the role of FK506 binding protein 51 (FKBP51) in human endometrial adenocarcinoma progression. Immunohistochemical analysis showed decreased FKBP51 expression in endometrial adenocarcinoma tissues. Moreover, higher FKBP51 expression was observed in the normal secretory phase than in proliferative-phase endometrial tissues. FKBP51-shRNA transfected KLE cells showed high Ser473-phospho Akt with decreased p21 and p27 levels, which promoted S-G_2_/M phase cell cycle progression and proliferation. Conversely, FKBP51 overexpressing Ishikawa cells showed low Ser473-phospho Akt, which led to increased p21 and p27 levels and, in turn, G_0_/G_1_ cell cycle arrest and decreased cell proliferation. FKBP51 overexpression in progesterone receptor-positive Ishikawa cells sensitized them to medroxyprogesterone acetate (MPA; progestin) treatment by repressing Akt signaling. Conversely, FKBP51-shRNA knockdown in RL95-2 cells attenuated progestin sensitivity. These findings indicate FKBP51 inhibits cell proliferation and promotes progestin sensitivity in endometrial adenocarcinoma by decreasing Akt signaling.

## INTRODUCTION

FK506 binding protein 51 (FKBP51) is a 51-kDa protein that belongs to a family of FK506-binding proteins and contains FK506 binding and tetratricopeptide repeat (TPR) domains [1, 2]. Although human FKBP51 has two consecutive FK506 binding domains (FK1 and FK2), only FK1 interacts with immunosuppressants and possesses the peptidylprolyl isomerase (PPIase) activity, thereby acting as an NF-κB activator [3]. Both FK domains are involved in Akt binding, whereas the three-unit repeat of the TPR domain in the C-terminus forms super-chaperone complexes with heat shock proteins and steroid receptors and modulates steroid receptor activity [4–7].

FKBP51 is robustly stimulated by steroid hormones such as progesterone, glucocorticoid and androgen, but not estrogen [8]. FKBP51 is expressed in many normal human tissues and overexpressed or downregulated in various human cancers [9]. FKBP51 is overexpressed in prostate cancer and gliomas and promotes tumorigenesis and chemoresistance through steroid receptor or NF-κB [10–12]. Meanwhile, FKBP51 is downregulated in pancreatic cancer and impairs cellular responses to chemotherapy through the PI3K/AKT signaling pathway [12–16].

Endometrial adenocarcinoma is the most common gynecologic malignancy in women, especially from western countries. There has been no improvement in survival rates over the past four decades, but, there is a constant increase in newly diagnosed patients [17–19]. The most common form of endometrial cancer is Type I endometrial adenocarcinomas. In normal endometrium, estrogen promotes cell growth whereas progesterone counteracts the effect of estrogen by promoting cell differentiation. In endometrial hyperplasia and/or carcinoma, cells are exposed to excess estrogen and relatively insufficient progesterone [20–22]. Treatment for type I endometrial adenocarcinoma involves radical surgery combined with adjuvant radiation and chemotherapy. Currently, the progestin, medroxyprogesterone acetate (MPA), which is also a frontline drug for fertility-preservation has been used for recurrent and advanced endometrial carcinomas when surgical intervention is not clinically feasible [23–25]. However, deficient progesterone receptor (PR) correlates with poor prognosis and response to progestin therapy; gradual loss of PR due to prolonged progestin treatment is also a major hurdle for hormone therapy [26]. Therefore, novel approaches are necessary to improve progestin sensitivity in endometrial adenocarcinoma patients.

Endometrial adenocarcinoma is a sex hormone-related disease, which is characterized by high levels of estrogen that outweigh progesterone. FKBP51 regulates the activity of sex-hormone receptors and is induced by progesterone through PR binding [27, 28]. Therefore, in this study, we investigated the role of FKBP51 in endometrial adenocarcinoma cell growth and progestin resistance.

## RESULTS

### Low FKBP51 expression in human endometrial adenocarcinoma tissues

**Figure 1:**
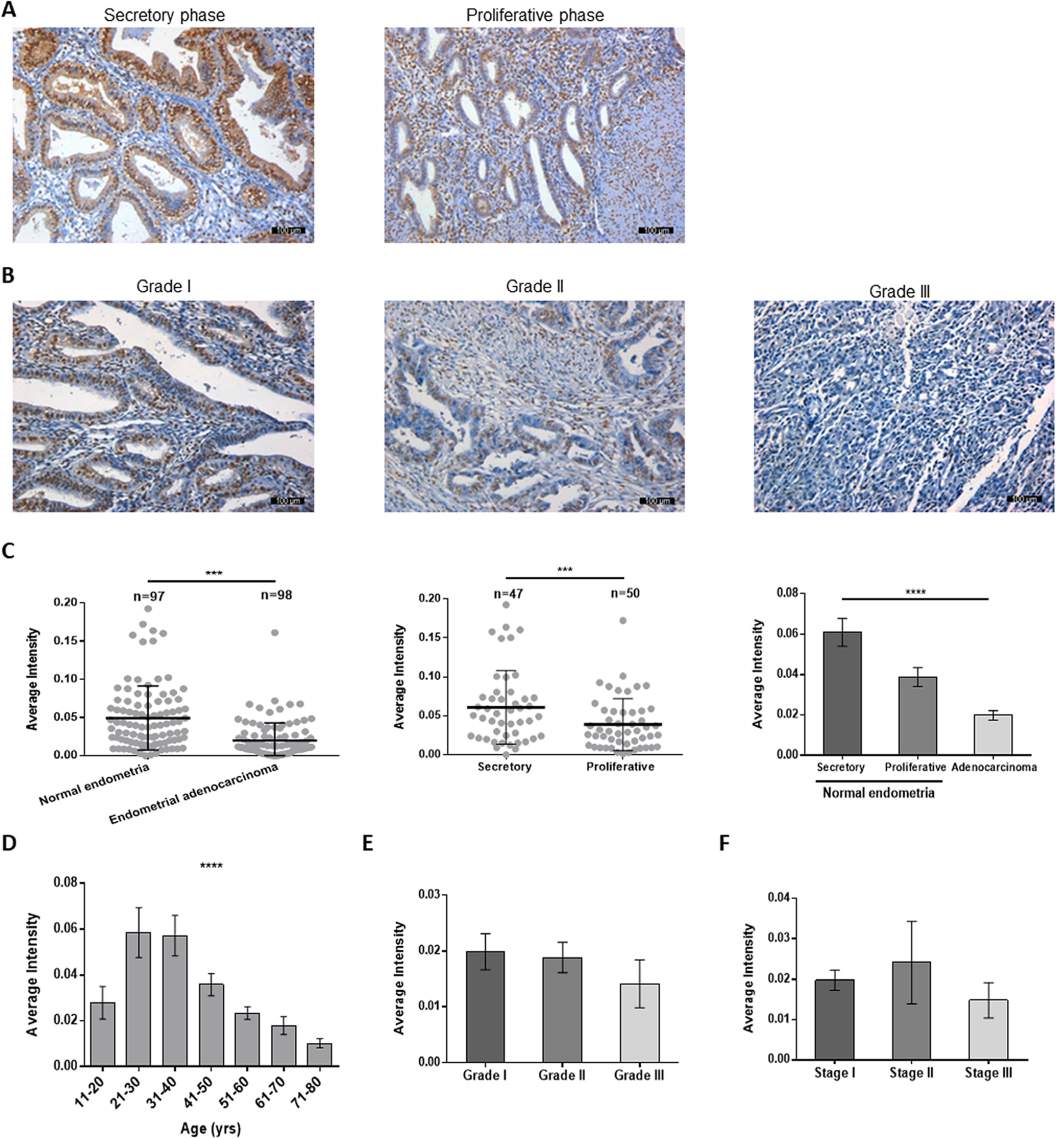
FKBP51 expression in normal endometrial and endometrial adenocarcinoma tissues (**A**) Representative images (magnification, 200×) of FKBP51-IHC stained normal endometrial tissues are shown. (**B**) FKBP51 expression in endometrial adenocarcinoma tissues (magnification, 200×). As observed, FKBP51 levels are lower in endometrial adenocarcinoma than in normal endometrium tissues. FKBP51 expression is higher in secretory phase endometrium than in proliferative phase. (**C**) The immunohistochemical intensity of FKBP51 were calculated in normal endometrial and endometrial adenocarcinoma tissues. (**D**) The immunohistochemical intensity of FKBP51 in tissues from different ages were calculated. (**E**) The differences among grades I, II and III endometrial adenocarcinomas were not statistically significant. (**F**) The differences among stages I, II and III endometrial adenocarcinomas were not statistically significant. These plots show mean Integrated optical density (IOD) of immunohistochemically stained normal and endometrial adenocarcinoma tissues for FKBP51. Each stained sample was measured and analyzed using Image-Pro Plus and SPSS. Data was shown as the mean ± SD. ***p < 0.001, ****p < 0.0001. Scale bar: 100 μm.

IHC analysis showed nuclear and cytoplasmic FKBP51 staining in both normal and cancerous endometrial tissues (Figure 1A–1B). FKBP51 levels were lower in endometrial adenocarcinoma specimens than in normal endometrium samples (Figure 1A–1C). Among normal endometrial tissues, FKBP51 expression was lower in proliferative phase than in the secretory phase (Figure 1A, 1C). Moreover, FKBP51 expression decreased with age in adulthood and lower FKBP51 levels were observed in older women (Table 1, Figure 1D), but, there was no correlation with the pathological grade or clinical stage (Figure 1E–1FE, Table 1). These data implied that FKBP51 suppressed endometrial cell growth.

**Table 1:** Distribution of patients and tissue characteristics

Characteristics	Female			FKBP51 ($\bar{x}\pm S$)	*P*-value
	Cases (*n* = 98)	Controls (*n* = 97)	Total (*n* = 195)		
Age (y)					< 0.0001
11–20	-	3	3	0.03 ± 0.01	
21–30	-	25	25	0.06 ± 0.05	
31–40	7	23	30	0.06 ± 0.05	
41–50	14	26	40	0.04 ± 0.03	
51–60	49	18	67	0.02 ± 0.02	
61–70	21	2	23	0.02 ± 0.02	
71–80	7	-	7	0.01 ± 0.01	
Median (y)	56.5 (y)	38 (y)	50 (y)		
Mini-Maxi (y)	31–78 (y)	12–64 (y)	12–78 (y)		
Histological grade					0.49
I	34	-	34	0.02 ± 0.02	
II	46	-	46	0.02 ± 0.02	
III	18	-	18	0.02 ± 0.04	
TNM stage					0.8
I	90	-	90	0.02 ± 0.02	
II	5	-	5	0.02 ± 0.02	
III	3	-	3	0.01 ± 0.01	
Menstrual cycle phase					0.0087
Proliferative phase	-	50	50	0.04 ± 0.03	
Secretory phase	-	47	47	0.06 ± 0.05	

### FKBP51 inhibits endometrial adenocarcinoma growth and cell cycle progression

**Figure 2:**
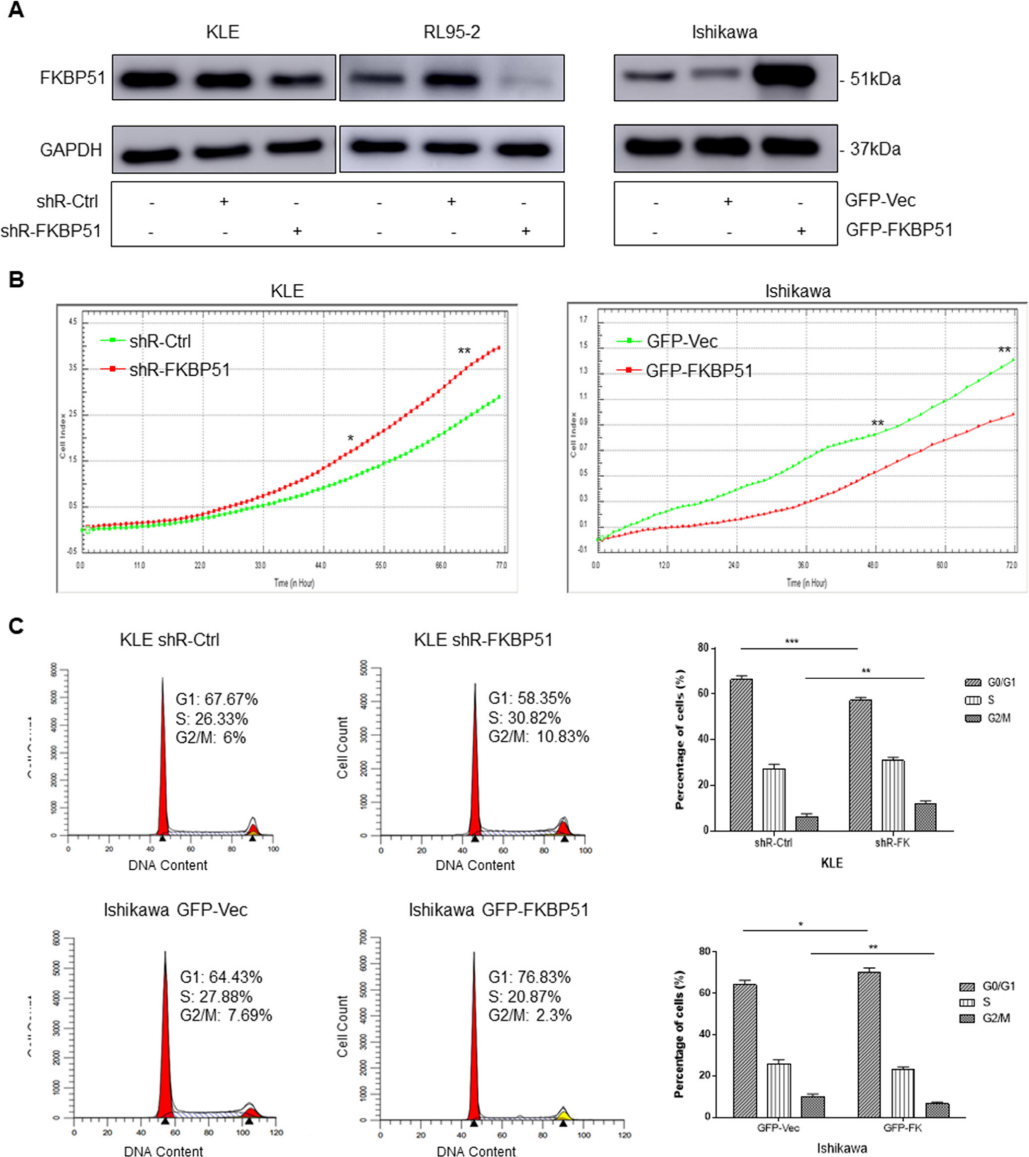
FKBP51 negatively regulates endometrial adenocarcinoma cell growth (**A**) Western blot analysis of FKBP51 expression in control- or FKBP51-shRNA transfected KLE and RL95-2 cells as well as control and FKBP51 overexpressing Ishikawa cells is shown. (**B**) RTCA analysis of cell growth parameters in control- or FKBP51-shRNA transfected KLE cells as well as control and FKBP51 overexpressing Ishikawa cells is shown as monitored over 72 h by xCELLigence SP system. *P* values were analyzed by using repeated-measurement ANOVA. (**C**) FACS analysis of PI stained control- or FKBP51-shRNA transfected KLE cells as well as control and FKBP51 overexpressing Ishikawa cells is shown. The cell cycle analysis shows that FKBP51 shRNA transfected KLE cells show lower G_0_/G_1_ phase cells and higher G_2_/M phase cells than in control KLE cells. Also, FKBP51 overexpressing Ishikawa cells show higher G_0_/G_1_ phase cells and lower G_2_/M phase cells than in control cells. This confirms that higher FKBP51 levels reduce endometrial adenocarcinoma cell growth by G_0_/G_1_ cell cycle arrest. Each experiment was performed in triplicate. *p < 0.05, **p < 0.01, ***p < 0.001.

FKBP51 function was analyzed in KLE and Ishikawa endometrial adenocarcinoma cell lines. We performed shRNA knock down of FKBP51 in KLE cells that expressed high FKBP51 protein and overexpressed FKBP51 in Ishikawa cells that showed low FKBP51 protein expression. Western blot analysis confirmed that FKBP51 was decreased in the shRNA transfected KLE cells and increased in Ishikawa cells infected with FKBP51 overexpressing lentiviral vector (Figure 2A).

Cell proliferation was increased in FKBP51-shRNA transfected KLE cells and decreased in FKBP51 overexpressing Ishikawa cells (Figure 2B). Cell cycle analysis demonstrated that FKBP51 overexpressing Ishikawa cells increased G_0_/G_1_ phase cells and decreased G_2_/M and S-G_2_/M phase cell numbers. In contrast, FKBP51-shRNA transfected KLE cells increased G_2_/M and S-G_2_/M phase cell numbers and decreased percent G_0_/G_1_ phase cells (Figure 2C). This demonstrated that high FKBP51 expression inhibited cell proliferation by inducing cell cycle arrest in endometrial adenocarcinoma.

### High or low FKBP51 levels do not alter endometrial adenocarcinoma cell apoptosis and invasiveness

**Figure 3:**
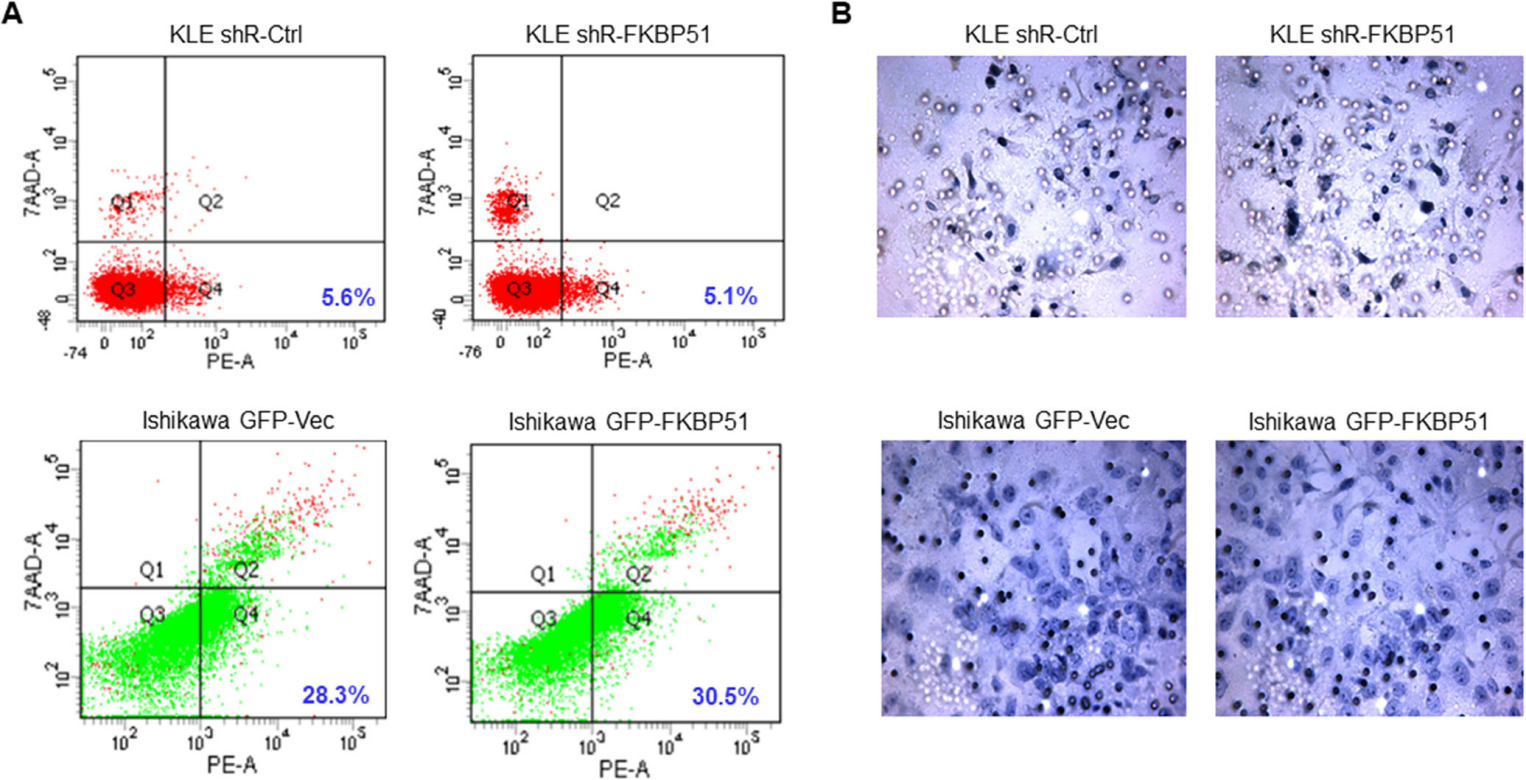
Apoptosis and cell invasion analyses of endometrial adenocarcinoma cells (**A**) FACS analysis to determine percent AnnexinV^+^7AAD^−^ (apoptotic) cells in KLE cells transfected with control or FKBP51 shRNA as well as control and FKBP51 overexpressing Ishikawa cells is shown. Note: *P* values were > 0.05; altered FKBP51 expression did not affect apoptosis rates in endometrial adenocarcinoma cells. (**B**) Transwell matrigel analysis showing invasion capacity of KLE cells transfected with control or FKBP51 shRNA as well as control and FKBP51 overexpressing Ishikawa cells is shown. Note: *P* values were > 0.05; altering FKBP51 expression did not affect invasiveness of endometrial adenocarcinoma cells.

Next, we analyzed if high or low FKBP51 expression altered cellular apoptosis and invasiveness of endometrial adenocarcinoma cells. Flow cytometry analyses showed that percent apoptotic cells (annexinV^+^ 7AAD^+^) were similar in both FKBP51-shRNA transfected KLE and FKBP51 overexpressing Ishikawa cells (Figure 3A). Further, Transwell matrigel assay showed that both FKBP51-shRNA transfected KLE and FKBP51 overexpressing Ishikawa cells demonstrated similar invasiveness (Figure 3B). This suggested that FKBP51 levels did not alter endometrial adenocarcinoma cell apoptosis or invasion.

### FKBP51 regulates Akt signaling in endometrial adenocarcinoma cells

**Figure 4:**
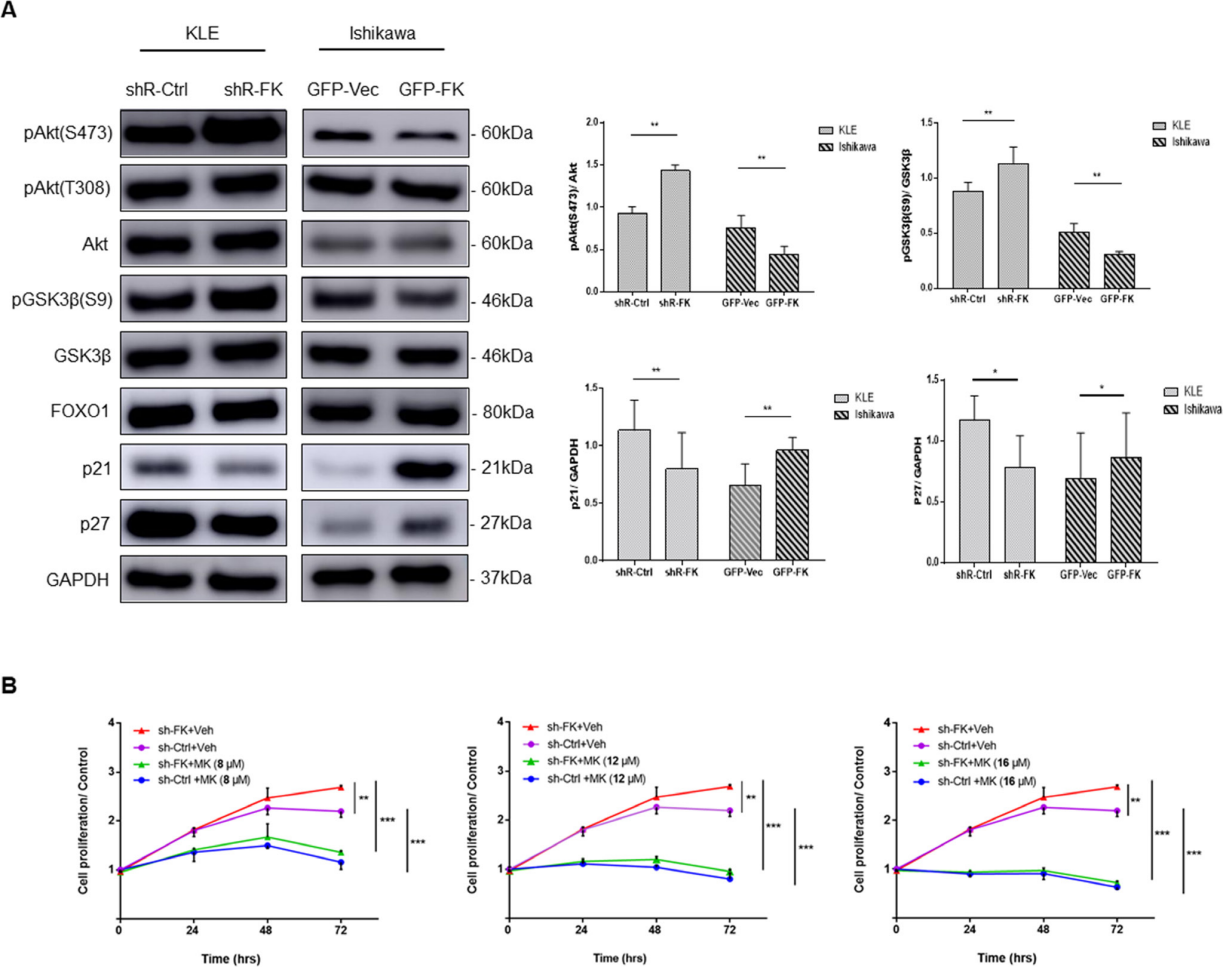
FKBP51 regulates endometrial adenocarcinoma cell growth by inhibiting Akt (**A**) Western blot analysis of total and phosphorylated (Ser473 and Thr308) Akt as well as its downstream targets, p21, p27, Foxo1 and phosphorylated (Ser9) GSK3β in KLE cells transfected with control or FKBP51 shRNA as well as control and FKBP51 overexpressing Ishikawa cells is shown. (**B**) CCK-8 assay of control- and FKBP51-shRNA transfected KLE cells treated with three concentrations (8, 12 and 16 μM) of MK-2206 or vehicle control (dimethyl sulfoxide) is shown. Each experiment was performed in triplicate. *p < 0.05, **p < 0.01.

Next, we investigated if FKBP51 inhibited endometrial adenocarcinoma cell growth by negatively regulating Akt. We observed Ser473-phospho Akt levels were increased in FKBP51-shRNA transfected KLE cells, but decreased in FKBP51 overexpressing Ishikawa cells (Figure 4A). However, Thr308-phospho Akt levels were not altered by FKBP51 knockdown or overexpression (Figure 4A). These results suggested that FKBP51 regulated Akt phosphorylation at Ser473.

To confirm changes in Akt signaling based on FKBP51 levels, we studied the status of the downstream Akt substrates, namely, GSK3β, FOXO1 and the cyclin-dependent kinase inhibitors (CDKIs) p21 and p27. FKBP51-shRNA transfected KLE cells showed low p21 and p27 as well as increased Ser9 phosphorylation of GSK3β (Figure 4A). In contrast, FKBP51 overexpressing Ishikawa cells showed high p21 and p27 levels and reduced Ser9 phosphorylation of GSK3β (Figure 4A). However, FOXO1 levels were unaffected by high or low FKBP51 levels (Figure 4A). This suggested that FKBP51 negatively regulated Akt phosphorylation in endometrial adenocarcinoma cells.

Further, MK-2206, a selective Akt inhibitor abolished FKBP51-shRNA induced cell proliferation in a dose-dependent manner (Figure 4B). This confirmed that FKBP51 inhibited endometrial adenocarcinoma cell proliferation via Akt signaling.

### FKBP51 increased endometrial adenocarcinoma cell sensitivity to MPA treatment

**Figure 5:**
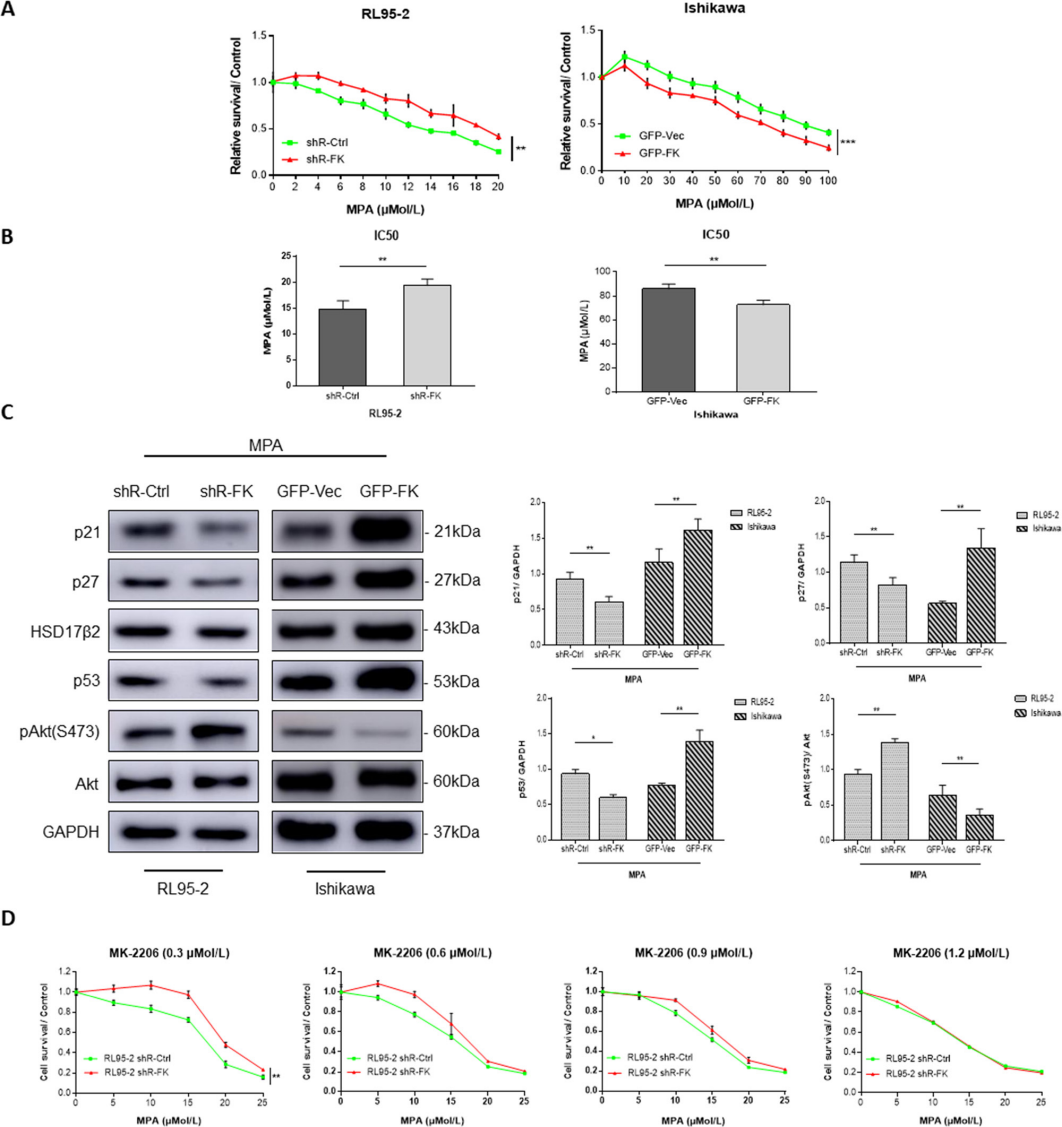
FKBP51 modulates MPA sensitivity by inhibiting Akt (**A**) CCK-8 assay of PR-positive control- or FKBP51-shRNA transfected RL95-2 cells as well as control and FKBP51 overexpressing Ishikawa cells treated with increasing concentrations (2–20 μM, 10–100 μM) of MPA or vehicle control (dimethyl sulfoxide) for 48 h is shown. (**B**) IC50 demonstrating MPA sensitivity of MPA-treated control- or FKBP51-shRNA transfected RL95-2 cells as well as control and FKBP51 overexpressing Ishikawa cells are shown as mean ± SD (*P* < 0.01). (**C**) Western blot analysis of MPA-treated (20 or 80 μM) control- or FKBP51-shRNA transfected RL95-2 cells as well as control and FKBP51 overexpressing Ishikawa cells show the levels of p21, p27, HSD17β2, p53 proteins in addition to total and Ser473 phospho- Akt. (**D**) MPA sensitivity assay of control- and FKBP51-shRNA transfected RL95-2 cells treated with four increasing concentrations (0.3, 0.6, 0.9 and 1.2 μM) of MK-2206 is shown. Each experiment was performed in triplicate. *p < 0.05, **p < 0.01, ***p < 0.001.

Next, we used PR-positive cell lines, Ishikawa and RL95-2 to examine if FKBP51 levels determined endometrial adenocarcinoma cell responses to MPA treatment. We observed that overexpression of FKBP51 sensitized Ishikawa cells to MPA, whereas FKBP51 shRNA knockdown in RL95-2 cells increased resistance to MPA (Figure 5A–5B).

We further examined if FKBP51 levels affected progesterone-related proteins including p21, p27, p53 and 17β-hydroxysteroid dehydrogenase type 2 (17-HSDβ2), which are upregulated by progesterone (or MPA) and involved in cell growth suppression [25]. We found that knockdown of FKBP51 in RL95-2 cells decreased p21, p27 and p53 levels upon MPA treatment, coinciding with increased Akt activity. In contrast, FKBP51 overexpression in Ishikawa cells increased p21, p27 and p53 expression upon MPA treatment, coinciding with decreased Akt activity (Figure 5C).

To determine if Akt signaling pathway was related to FKBP51-shRNA induced endometrial adenocarcinoma cell resistance to MPA, we treated RL95-2 cells with increasing concentrations of MPA for 48 hrs in the presence of four different concentrations of MK-2206, and analyzed MPA sensitivity. We observed that FKBP51-shRNA induced MPA resistance was abolished by MK-2206 treatment in a dose-dependent manner in RL95-2 cells (Figure 5D). This demonstrated that FKBP51 increased MPA sensitivity of endometrial adenocarcinoma cells by inhibiting AKT phosphorylation.

## DISCUSSION

FKBP51 was originally identified as a component of steroid receptor complexes [29]. Recently, the role of FKBP51 in regulating tumorigenesis and tumor response to chemotherapy has been recognized in some cancers [10–12]. However, its role in human endometrial adenocarcinoma is not known. Therefore, we investigated its role using multiple endometrial adenocarcinoma cell lines that express high or low FKBP51. IHC analysis showed lower FKBP51 expression in endometrial tissues from endometrial adenocarcinomas and normal-proliferative phase as well as elderly women than in endometrial tissues from normal endometria and normal-secretory phase or younger subjects. This was similar to previous studies that showed lower progesterone levels in endometrial tissues from normal proliferative-phase and aged women as well as endometrial adenocarcinomas. Also, previous studies showed that progesterone induced FKBP51 [30, 31]. FKBP51 downregulation increased cell proliferation and cell cycle progression, whereas its overexpression inhibited cell proliferation by inducing cell cycle arrest.

Our study also demonstrated that FKBP51 knockdown increased Akt phosphorylation at Ser473, thereby decreasing the expression of p21 and p27, which are cell cycle inhibitors. In contrast, FKBP51 overexpression decreased Ser473-phospho Akt that increased p21 and p27 expression. Moreover, AKT inhibitor MK-2206 abolished increased cell proliferation in FKBP51 knockdown cells. These results confirmed that high FKBP51 levels decreased endometrial adenocarcinoma progression by inhibiting Akt.

The AKT/protein kinase B (PKB) signaling pathway is constitutively activated in many tumor types [32, 33]. It regulates tumorigenesis and chemoresistance in many different cancers [34, 35]. Phosphorylation at Thr308 and Ser473 results in fully activation of Akt [32, 36]. FKBP51 is a scaffolding protein that specifically increases PHLPP-mediated dephosphorylation of AKT at Ser473 [4, 37]. In this study, we showed that FKBP51 decreased phosphorylation of Akt Ser473 and not Akt Thr308 in endometrial adenocarcinomas.

Cyclin-dependent kinase (CDK) complexes drive cell cycle progression. They can be inactivated by CDK inhibitors (CDKIs), such as p21^Waf1/Cip1^ and p27^Kip1^, which suppress the activity of CDK complexes and contribute to G_1_ phase cell cycle arrest [38, 39]. This correlates with our findings that overexpression of FKBP51 induced G_0_/G_1_ cell cycle arrest due to increased p21 and p27. Both, p21 and p27 are induced by MPA resulting in endometrial cancer growth inhibition [40]. Therefore, p21 and p27 have been used as biomarkers to evaluate MPA effectiveness in endometrial carcinomas [41]. Recent studies have shown that PI3K/AKT pathway hyperactivation by mutated tumor-suppressor genes could also be involved in progestin resistance [42]. This implies that FKBP51 may modulate cellular response to MPA through Akt pathway since p21 and p27 are downstream substrates of Akt. In our work, FKBP51 overexpression sensitized cancer cells to MPA treatment, while knockdown of FKBP51 decreased response to MPA. Moreover, MK-2206 abolished the FKBP51 knockdown-induced cell resistant to MPA in a dose-dependent manner, confirming that FKBP51 increased cell response to MPA treatment through Akt inhibition. This inhibitory effect led to improved MPA sensitivity by inducing p21 and p27 expression. Mutations in PTEN and PI3KCA genes are often observed in endometrial precancerous hyperplastic and cancer lesions resulting in constitutive PI3K/AKT pathway activation [42, 43]. Therefore, our study suggests that hyperactive Akt induces cell growth and resistance to hormone treatment, which could potentially be reversed by targeting FKBP51 expression in combination with AKT inhibitors.

In conclusion, our study demonstrates that FKBP51 is involved in endometrial adenocarcinoma inhibition as well as hormone-therapy sensitization by modulating Akt, thereby indicating therapeutic value.

## MATERIALS AND METHODS

### Cell lines, cell culture and reagents

The endometrial adenocarcinoma cell lines KLE, RL95-2 and Ishikawa were purchased from China Type Culture Collection (Shanghai, China) and BeNa Culture Collection (Beijing, China). Cells were grown in RPMI 1640 (Life Technologies, Inc. Grand Island, NY, USA) supplemented with 10% FBS (charcoal-stripped FBS when treated with MPA) at 37°C in a humidified atmosphere of 5% CO_2_. FBS was purchased from Life Technologies, Inc. (Grand Island, NY, USA), whereas charcoal-stripped FBS was purchased from BioInd Biological Industries, Inc. (Cromwell, CT, USA). MPA was purchased from Tokyo Chemical Industry Co., Ltd. (Tokyo, Japan). MK-2206 was obtained from Selleck chemicals, LLC. (Houston, TX, USA).

### Patient samples

Endometrial tissue microarrays that included adenocarcinomas and normal tissues were purchased from Cybrdi, Shanxi ChaoYing Biotechnology Company, Ltd. (Shanxi, China). A subset of the normal endometrial tissues was obtained from the Pathology Department of Provincial Hospital affiliated to Shandong University. Samples were collected after obtaining informed consent from the patients. The study methodology was according to Declaration of Helsinki guidelines. All adenocarcinoma samples were diagnosed and evaluated according to the International Federation of Gynecology Oncology (FIGO) criteria. In total, 195 endometrium samples were studied, including 98 endometrial adenocarcinoma tissue samples and 97 normal endometrium samples. The normal endometrium tissues came from the uteruses of hysteromyoma patients after hysterectomy.

### Immunohistochemistry

FKBP51 expression in endometrial tissues was determined by immunohistochemistry (IHC). Endometrial tissues were embedded in paraffin and 4 μm-thick sections were cut. The specimens were treated with 3% hydrogen peroxide in methanol to inhibit the endogenous peroxidases. Then, they were incubated overnight with primary antibody (anti-FKBP51; sc-13983; 1:25 dilution) at 4°C followed by staining with the Elivision™ plus kit (9902; Maxim Biotechnology, Rockville, MD, USA) and diaminobenzidine solution (Maxim Biotechnology).

### FKBP51 overexpressing plasmids and short interfering RNA

Plasmids encoding human FKBP51 shRNA (SC-35380-SH) and scramble shRNA were purchased from Santa Cruz Biotechnology, Inc. A human FKBP51 shRNA plasmid (SC-35380-SH) with a puromycin resistance gene for selection was designed to knockdown FKBP51 expression. The KLE and RL95-2 cells (2 × 10^5^) were seeded in a six-well plate and grown overnight. The next day, the medium was replaced with transfection medium containing the shRNA plasmid for 5–7 hrs followed by 2× normal growth medium for an additional 48 h. Then, the transfection medium was replaced by fresh medium containing 4 μg/ml puromycin (Sigma-Aldrich, St. Louis, MO, USA). Multiple positive transfectants were obtained after 3–5 days of puromycin selection.

### Lentivirus infection

The human FKBP51 gene was amplified and reverse transcribed to synthesize a cDNA. The FKBP51 cDNA was cloned into the GV303 lentiviral expression vector (GeneChem, Shanghai, China). Ishikawa cells (5 × 10^3^) were seeded in a 96-well plate overnight. Then, cells were infected with 1 × 10^6^ units of the recombinant lentiviral vector containing wild-type human FKBP51 or randomized flanking control sequences. The ratio of virus-infected cells, which were GFP positive, was checked by fluorescence microscope after 72 h of infection.

### Western blotting

Cells were lysed on ice with RIPA Lysis and Extraction Buffer (Thermo Fisher Scientific, USA) supplemented with protease and phosphatase inhibitors (Roche, Basel, Switzerland). Total extracted proteins were separated by electrophoresis with 10% SDS–polyacrylamide gels and transferred onto PVDF membranes (Millipore, Billerica, MA, USA). Then, the membranes were blocked with 5% skinned milk followed by incubation with primary antibodies overnight at 4^°^C. This was followed by incubation with secondary antibodies for 1 h at room temperature. The blots were washed with 1XTBST thrice followed by development with ECL. The relative amounts of various proteins were determined in relation to GAPDH as control. Antibodies to FKBP51 and GAPDH were purchased from Santa Cruz Biotechnology, Inc. (Santa Cruz, CA, USA). Antibodies against Akt, phospho-Akt (Ser473), phospho-Akt (Thr308), FOXO1, p21, p27 and p53 were purchased from Cell Signaling Technology, Inc. (Danvers, MA, USA). Antibodies to GSK3β, phospho-GSK3β (Ser9) and HSD17β2 were purchased from Abcam (Cambridge, MA, USA).

### Cell proliferation analysis

5 × 10^3^ cells were seeded in a 16-well E-plate (Roche) and cultured in a 5% CO_2_ incubator at 37°C. Cell growth was continuously monitored for 72 h by xCELLigence SP system, which used the Real Time Cellular Analysis (RTCA) technology developed by ACEA Biosciences, Inc. (San Diego, CA, USA). The xCELLigence^®^ RTCA DP instrument (Roche) was used to dynamically monitor proliferation and cell viability in a label-free manner. Data were tabulated and analyzed using the specialized RTCA software.

### Cell cycle assay

Cell cycle assay was performed with the Cycletest^TM^ Plus DNA Kit (340242; BD Biosciences, San Jose, CA, USA). 1.5 × 10^5^ cells were washed with Buffer solution and resuspended in solutions A and B to digest cell membrane, cytoskeleton and RNA. Then, the cells were incubated with solution C containing propidium iodide (PI). The cells were analyzed in a flow cytometer (BD Biosciences, San Jose, CA, USA) and the percentages of G_0_/G_1_, S and G_2_/M phase cells were analyzed by the ModFit LT software (Verity Software House Inc., Topsham, ME, USA).

### Apoptosis assay

Cells were washed with PBS and resuspended in AnnexinV- binding buffer. Then, 5 μl PE Annexin-V and 7-Amino-Actinomycin (7-AAD) was added to each sample and incubated for 15 min. The total numbers of AnnexinV^+^ 7AAD^−^ apoptotic cells were determined in a flow cytometer (Becton Dickinson, Franklin Lakes, NJ, USA).

### Transwell invasion assay

1.5 × 10^5^ cells in serum-free medium (200 μl) were added to the upper compartment of a Matrigel invasion chamber. The lower compartment was filled with 600 μl culture medium supplemented with 10% FBS. The plate was incubated at 37°C for 24 h. The invading cells in the lower chamber were fixed with 4% paraformaldehyde, stained with hematoxylin and counted.

### Progestin sensitivity assay

Cellular response to MPA treatment was examined by the Cell Counting Kit-8 (CCK8) obtained from Dojindo Molecular Technologies, Inc. (Shanghai, China). Cells were seeded in 96-well plates and grown for 24 h before treatment with increasing concentrations of MPA or 0.1% dimethylsulfoxide (DMSO, Sigma-Aldrich) as vehicle group. After 48 h incubation, the CCK-8 assay was performed according to manusfacturer's conditions by reading absorbance at 450 nm in a automated microplate reader.

### Statistical analyses

The data were analyzed with SPSS software (SPSS Inc., Chicago, IL, USA). Student's *t* test and one-way ANOVA was used to determine statistical differences between groups. *P* < 0.05 was considered statistically significant. All experiments were performed in triplicates.

## Abbreviations

CDKIs: Cyclin-dependent Kinase inhibitors
FKBPs: FK506 Binding Proteins
FOXO1: Forkhead box protein O1
GSK3β: Glycogen Synthase Kinase 3 beta
17-HSDβ2: 17β-Hydroxysteroid Dehydrogenase type 2
MPA: Medroxyprogesterone Acetate
PHLPP: PH domain and Leucine rich repeat Protein Phosphatases
PPIase: Peptidylprolyl Isomerase
PR: Progesterone Receptor
shRNA: short-hairpin RNA
